# Association between peripheral eosinophilia, JESREC score, and olfactory dysfunction in patients with chronic rhinosinusitis

**DOI:** 10.3389/fimmu.2024.1334656

**Published:** 2024-01-24

**Authors:** Ling Zhang, Haifeng Li, Tao Wang, Zhu Wang, Yang Wu, Siquan Guo, Wenjing Li, Yeru Zhou, Haixiang Xue, Jianqiang You

**Affiliations:** Department of Otorhinolaryngology, The First People’s Hospital of Changzhou, The Third Affiliated Hospital of Soochow University, Soochow University, Changzhou, China

**Keywords:** chronic rhinosinusitis, olfactory dysfunction, peripheral eosinophilia, JESREC score, eosinophilic chronic rhinosinusitis

## Abstract

**Objective:**

The purpose was to evaluate the relationship between peripheral eosinophilia, Japan Epidemiological Survey of Refractory Eosinophilic Chronic Rhinosinusitis (JESREC) score, and olfactory dysfunction in chronic rhinosinusitis (CRS) patients and to explore the accuracy and specific cut points of the JESREC score in predicting olfactory dysfunction.

**Methods:**

In this cross-sectional, retrospective study, olfactory function was assessed by the Sniffin’ Sticks 12-item test and multivariate logistic regression analyses were carried out. Receiver operating characteristic curves were plotted to derive accuracy and cutoff values for the JESREC scores of the olfactory dysfunction criterion.

**Results:**

A total of 354 patients [mean (SD) age, 50.0 (14.9) years; 41.8% women] were included in the final analysis. The prevalence of olfactory dysfunction was 46.3%. Individuals who had olfactory dysfunction were more likely to be male (64.6% vs. 52.6%), have eosinophilic chronic rhinosinusitis (ECRS) (39.0% vs. 7.9%), have a longer course of CRS (2.3 years vs. 1.5 years), have higher JESREC scores (8.5 vs. 4.5), and have higher proportions of nasal polyps (78.7% vs. 18.9%) and peripheral eosinophilia (3.3% vs. 1.4%). In logistic analysis, the percentage of eosinophils (1.25, 1.13–1.37), JESREC score (1.31, 1.22–1.40), bilateral lesion (2.06, 1.25–3.41), nasal polyps (15.83, 9.23–27.16), CT shadow (2.73, 1.69–4.43), and ECRS (6.86, 3.68–12.80) were associated with olfactory dysfunction in CRS patients after controlling for covariates, while peripheral neutrophils were not significant. In addition, the area under the curve was 0.778 and the cutoff value for JESREC score for olfactory dysfunction was defined as 5.5.

**Conclusions:**

Peripheral eosinophilia and high JESREC scores were significantly associated with the risk of olfactory dysfunction in CRS patients, and special attention should be paid to patients with a JESREC score ≥6.

## Introduction

Chronic rhinosinusitis (CRS) is a common chronic inflammatory disease of the nasal cavity and paranasal sinuses, which is often accompanied by olfactory dysfunction (1). Studies have shown that the incidence of olfactory dysfunction secondary to CRS is between 56% and 74%, with significant effects on the quality of life, including effects on diet, communication, and emotional wellbeing and loss of perception of dangerous odors, such as gases and toxic and harmful gases. However, the underlying mechanism remains elusive (2–5).

Eosinophils are key players in the pathogenesis of allergic diseases and are increasingly recognized to play a role in the pathogenesis of CRS through the release of inflammatory mediators and tissue damage (6–8). Elevated levels of eosinophils have been observed in the nasal polyps and mucosa of patients with CRS, and their activation and release of cytokines and other mediators have been implicated in the development of CRS-associated olfactory dysfunction (9, 10). Peripheral eosinophilia is a common feature of CRS, with elevated eosinophil counts present in up to 40% of patients. Elevated peripheral eosinophil counts have been associated with more severe CRS symptoms and poorer treatment outcomes (11–13), but their relationship with olfactory dysfunction has not been widely studied. In addition, based on clinical data, the Japan Epidemiological Survey of Refractory Eosinophilic Chronic Rhinosinusitis (JESREC) study divided CRS into two subtypes, eosinophilic chronic rhinosinusitis (ECRS) and non-ECRS, which promotes precise research. Previous studies have confirmed that the JESREC scoring system was significantly correlated with prognosis (14), but few studies have focused on the relationship between JESREC score and olfactory dysfunction, and no studies have identified specific cut points. Considering the easy availability and operability of the JESREC scoring system in clinical application, it is very important and urgent to clarify the relationship between JESREC score and olfactory dysfunction and the specific cutoff point of the JESREC score in predicting olfactory dysfunction among CRS patients.

Based on these observations, the aim of our study was to investigate the potential relationship between peripheral eosinophilia, JESREC score, and olfactory dysfunction in patients with CRS. In addition, receiver operating characteristic curves were plotted to derive accuracy and cutoff values for the JESREC scores of the olfactory dysfunction criterion.

## Materials and methods

### Study population and design

The study population is composed of the 354 CRS patients who were assessed at the Third Affiliated Hospital of Soochow University (Changzhou, China) between March 2021 and September 2022. As described in our previous research (15), they were all Han Chinese, and none of them were infected with COVID-19. The study was approved by the Ethics Committee of Third Affiliated Hospital of Soochow University (2022CL070), and due to the use of anonymous data and the lack of any intervention, informed consent was not required.

The inclusion criteria were as follows: 1) adult patients aged over 18 years; 2) complete clinical data, including detailed medical records and nasal endoscopy, laboratory, and imaging examinations; and 3) conformity to the European Position Paper on Rhinosinusitis and Nasal Polyps (EPOS) 2012 diagnostic criteria of CRS (16). The exclusion criteria were as follows: 1) history of medical treatment or surgery for CRS, 2) asthma and liver diseases, 3) nasal diseases that may affect olfactory sensitivity, and 4) taking glucocorticoids or hepatotoxic drugs.

### Data collection and covariates

Data collection was performed by medical staff in the department of otolaryngology according to a standard protocol when the patient first came to our hospital. Detailed information on demographic characteristics, medical records, and laboratory testing was obtained. Current drinkers were defined as those who were drinking or had stopped drinking for less than 6 months, and smokers were defined as those who were currently smoking or had stopped smoking for less than 6 months. The duration of CRS (unit: year) was defined as years between the first diagnosis and baseline evaluation in a diagnosed patient. Nasal polyps were identified by nasal endoscopy or sinus CT. Peripheral blood collection and complete blood count were performed in the central clinical laboratory of Third Affiliated Hospital of Soochow University by automated analyzers.

The criteria of the JESREC score (14) were as follows: 1) disease side: both sides vs. one side (3 vs. 0); 2) nasal polyps: presence vs. absence (2 vs. 0); 3) CT shadow: ethmoid ≥ maxillary: positive vs. negative (2 vs. 0); and 4) percentage of eosinophils of peripheral blood: ≤2 vs. >2 to ≤5 vs. >5 to ≤10 vs. >10 (0 vs. 4 vs. 8 vs. 10). A JESREC score ≥11 indicated ECRS and <11 indicated non-ECRS.

### Assessment of olfactory dysfunction

Olfactory function was assessed by the Sniffin’ Sticks 12-item test (SST-12). Briefly, participants were asked to smell for 3 to 4 s in turn and identify correct odors using a multiple-choice format. Correctly identified odorants were assigned 1 point, and the total of the SST-12 score ranges from 0 to 12. An SST-12 score <11 indicated olfactory dysfunction (17, 18).

### Statistical analysis

IBM SPSS Statistics 24.0 (SPSS Inc., Chicago, IL, United States) was used for all statistical analyses. Continuous variables were expressed as mean with SD, and categorical variables were presented as frequency (percentage). The characteristics of the participants were separated by olfactory function, and comparison between the two groups was performed by two-tailed unpaired t-tests or chi-squared tests (χ^2^-test) or Wilcoxon signed-rank tests. Multivariate logistic regression analyses were carried out to examine the associations of eosinophils of peripheral blood, JESREC score, and CRS subtype with the risk of olfactory dysfunction. Odds ratios (ORs) were estimated from two models: model 1 was adjusted for age and sex; model 2 was further adjusted for smoking and alcohol consumption. In addition, to derive cutoff values for the JESREC scores of olfactory dysfunction criterion, we constructed receiver operating characteristic (ROC) curves. Significance was accepted at P < 0.05.

## Results

### General characteristics

**Table 1** shows the general characteristics of the participants. A total of 354 patients [mean (SD) age, 50.0 (14.9) years; 41.8% women; mean (SD) duration of CRS, 1.8 (2.9) years] were included in the final analysis. Compared with patients with normal olfactory function, individuals who had olfactory dysfunction were more likely to be male (64.6% vs. 52.6%), have ECRS (39.0% vs. 7.9%), have a longer course of CRS (2.3 years vs. 1.5 years), have higher JESREC scores (8.5 vs. 4.5), and have higher proportions of nasal polyps (78.7% vs. 18.9%), bilateral lesion (79.9% vs. 64.2%), and peripheral blood eosinophils (3.3% vs. 1.4%).

**Table 1:** General characteristics of participants.

	CRS participants			*P*-value
	Total	With olfactory dysfunction	With normal olfactory	
Male/female	206/148	106/58	100/90	0.023*
Age of visit, years	50.0 ± 14.9	49.1 ± 15.4	50.8 ± 14.4	0.271
Duration of onset, years	1.8 ± 2.9	2.3 ± 3.0	1.5 ± 2.8	0.013*
Current smoker	36 (10.2%)	13 (7.9%)	23 (12.1%)	0.195
Alcohol drinker	31 (8.8%)	14 (8.5%)	17 (8.9%)	0.892
Body mass index, kg/m^2^	24.2 ± 3.5	24.1 ± 3.5	24.3 ± 3.5	0.696
Bilateral lesion	253 (71.4%)	131 (79.9%)	122 (64.2%)	<0.001*
JESREC score	6.3 ± 4.3	8.5 ± 4.2	4.5 ± 3.4	<0.001*
Eosinophilic chronic rhinosinusitis	79 (22.3%)	64 (39.0%)	15 (7.9%)	<0.001*
Nasal polyps	165 (46.6%)	129 (78.7%)	36 (18.9%)	<0.001*
CT shadow: ethmoid ≥ maxillary	233 (65.8%)	129 (78.7%)	104 (54.7%)	<0.001*
Peripheral blood				
Neutrophils, count × 10^9^/L	4.9 ± 2.6	4.7 ± 2.5	5.0 ± 2.6	0.167
Neutrophils, %	67.3 ± 12.8	66.6 ± 12.9	68.0 ± 12.6	0.251
Eosinophils, count × 10^7^/L	13.5 ± 22.8	19.6 ± 30.5	8.3 ± 10.6	<0.001*
Eosinophils, %	2.3 ± 3.5	3.3 ± 4.6	1.4 ± 1.8	<0.001*
Involvement of nasal sinuses				
Maxillary sinus	339 (95.8%)	155 (94.5%)	184 (96.8%)	0.279
Ethmoid sinus	288 (81.4%)	145 (88.4%)	143 (75.3%)	0.002*
Frontal sinus	171 (48.3%)	101 (61.6%)	70 (36.8%)	<0.001*
Sphenoid sinus	148 (41.8%)	95 (57.9%)	53 (27.9%)	<0.001*

Values are expressed as mean ± SE or n (%). *Significant at p < 0.05.

### Association between peripheral eosinophilia, JESREC score, and olfactory dysfunction

**Table 2** shows the multivariate logistic regression analysis with olfactory dysfunction among adult CRS patients. The adjusted OR for olfactory dysfunction was not significant with peripheral blood neutrophils, which were displayed as counts (0.94, 0.86–1.03) or percentages (0.99, 0.97–1.01). The JESREC score (1.31, 1.22–1.40) and its various components (bilateral lesion: 2.06, 1.25–3.41; nasal polyps: 15.83, 9.23–27.16; CT shadow: 2.73, 1.69–4.43; percentage of eosinophils of peripheral blood: 1.25, 1.13–1.37) were associated with olfactory dysfunction after adjusting for covariates. In addition, compared with non-ECRS, ECRS determined by the JESREC score was also risk factor of olfactory function (model 2, 6.86, 3.68–12.80).

**Table 2:** Multivariate logistic regression analysis with olfactory dysfunction as a dependent variable in CRS patients.

	Model 1	*P*-value	Model 2	*P*-value
Bilateral lesion	2.04 (1.24–3.35)	0.005	2.06 (1.25–3.41)	0.005
Nasal polyps	14.98 (8.85–25.35)	<0.001*	15.83 (9.23–27.16)	<0.001*
CT shadow: ethmoid ≥ maxillary	2.71 (1.68–4.38)	<0.001*	2.73 (1.69–4.43)	<0.001*
Peripheral blood				
Neutrophils, count	0.93 (0.85–1.01)	0.091	0.94 (0.86–1.03)	0.164
Neutrophils, %	0.99 (0.97–1.01)	0.198	0.99 (0.97–1.01)	0.255
Eosinophils, count	1.04 (1.02–1.06)	<0.001*	1.04 (1.02–1.06)	<0.001*
Eosinophils, %	1.26 (1.14–1.38)	<0.001*	1.25 (1.13–1.37)	<0.001*
JESREC score	1.31 (1.22–1.41)	<0.001*	1.31 (1.22–1.40)	<0.001*
Eosinophilic chronic rhinosinusitis	7.00 (3.77–13.00)	<0.001*	6.86 (3.68–12.80)	<0.001*

Values are expressed as odds ratio (95% confidence interval). *Significant at p < 0.05.

Model 1: adjusted for age, gender, and duration of CRS.

Model 2: adjusted for age, gender, duration of onset, smoking status, alcohol intake, and BMI.

**Figure 1:**
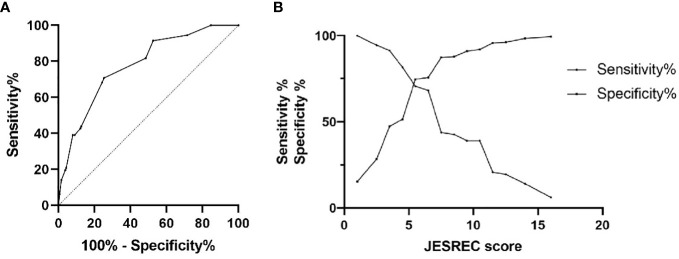
ROC curve analysis for JESREC score on olfactory dysfunction among adult CRS patients. **(A)** ROC curve for olfactory dysfunction: AUC was 0.778. **(B)** Sensitivity‐Specificity Plot: Cutoff value of JESREC score was 5.5, intersection point.

To clarify the specific cut points of JESREC score for predicting olfactory dysfunction in patients with CRS, the ROC curve was plotted (**Figure 1A**). The area under the curve (AUC) was 0.778, and the cutoff value for the JESREC score for olfactory dysfunction was defined as 5.5 (**Figure 1B**). If the JESREC score was 6 or higher, the case was more likely to have a decreased sense of smell, which requires attention and active treatment from doctors. Sensitivity and specificity were 70.7% and 74.7%, respectively.

## Discussion

This study indicated that peripheral eosinophilia and JESREC score were strongly associated with olfactory dysfunction among adult CRS patients. A JESREC score ≥6 should be a warning of the risk of olfactory dysfunction.

In the present study, 46.3% patients suffered from olfactory dysfunction, and among them, 64.6% were male, which was similar to a previous study (19, 20). In recent years, a growing number of studies have confirmed that CRS subtypes classified according to JESREC score and components of JESREC score, including nasal polyps, peripheral eosinophilia, and lesion location, were closely related with olfactory function (14, 21–23). Eosinophilia in peripheral blood and the nasal mucosa were common in CRS, especially in ECRS patients (24), and compared with CRS patients with normal olfactory function, patients with olfactory dysfunction had higher proportions of ECRS subtype and eosinophilia in our study, which was consistent with previous results (25, 26). Ahn et al. (27) reported that the olfactory score of ECRS patients was lower than that of non-ECRS patients. In addition, neutrophils, as the most common inflammatory cells in non-ECRS (28, 29), did not differ in the two groups in this study and multivariate logistic analysis also indicated that peripheral neutrophilia was not associated with olfactory dysfunction.

In the clinical analysis, logistic regression results revealed a risk relationship between peripheral eosinophilia; JESREC score; bilateral lesion; nasal polyps; CT shadow: ethmoid ≥ maxillary; and ECRS and olfactory dysfunction. It is important to emphasize that despite alcohol and tobacco consumption not demonstrating any statistical association in the bivariate analysis of this study, considering the potential effects of smoking and alcohol consumption on CRS patients in previous studies, we still included them in the analysis of model 2. Specifically, in our previous article analyzing the relationship between olfactory dysfunction and metabolic syndrome in CRS patients (15), alcohol and tobacco consumption were significantly different between the different groups. Furthermore, we also referred to models from other scholars’ studies (30–33), which also demonstrated the potential impact of smoking and alcohol consumption on CRS. Of course, it should be emphasized that the influence of smoking and alcohol consumption on CRS patients is still controversial, but considering the consistency of the model 1 and model 2 results in this study, we still show the results of the two models in the final result.

The mechanisms underlying CRS-associated olfactory loss are not fully understood, and the possible mechanisms are as follows: bilateral lesion and nasal polyps work by blocking the nasal cavity to prevent the odor molecules from reaching the olfactory region (34, 35); an increase in peripheral eosinophils is closely related to the percentage of infiltration of eosinophils in the sinuses, and numerous infiltrated eosinophils in the olfactory mucosa and nasal polyps may promote c-Jun N-terminal kinase pathway activation by secreting their stored granule proteins and cytokines, including interleukin 5 (IL-5), transforming growth factor α (TGF-α), and IL-2, leading to apoptosis and death of olfactory sensory neurons (36–38). In addition, previous studies have shown that the JESREC score can be used to distinguish between ECRS and non-ECRS and was useful for predicting CRS endotypes (14, 39). Our study was the first to describe a cutoff value for the JESREC score for olfactory dysfunction, which could assist doctors in more accurate prediction of long-term changes in olfaction among adult CRS patients and provide more targeted treatment.

Our study has several limitations. Firstly, limited by the cross-sectional study design, further research is needed to clarify the causal relationship between peripheral blood eosinophilia, JESREC score, and olfactory dysfunction. Secondly, all participants in this study were Han Chinese, and caution is still required in generalizing the conclusions to other ethnicities. Thirdly, being limited to a retrospective cross-sectional study and having incomplete clinical data, we lack information on skin testing or blood allergy testing that can reflect the atopic status of the patients, which may have potential impact on the results. In addition, although this article explored the potential mechanism of peripheral eosinophilia and JESREC score on olfactory decline in CRS patients, further research is needed on the specific pathophysiology.

In summary, peripheral eosinophilia and high JESREC scores were significantly associated with the risk of olfactory dysfunction in patients with chronic rhinosinusitis, and special attention should be paid to patients with JESREC score ≥6.

## Data availability statement

The raw data supporting the conclusions of this article will be made available by the authors, without undue reservation.

## Ethics statement

The studies involving humans were approved by the Ethics Committee of Third Affiliated Hospital of Soochow University. The studies were conducted in accordance with the local legislation and institutional requirements. Written informed consent for participation was not required from the participants or the participants’ legal guardians/next of kin because of anonymous data and the lack of any intervention.

## Author contributions

LZ: Conceptualization, Data curation, Formal analysis, Methodology, Validation, Visualization, Writing – original draft, Writing – review & editing. HL: Methodology, Software, Writing – original draft. TW: Data curation, Software, Validation, Writing – original draft. ZW: Formal Analysis, Project administration, Writing – original draft. YW: Funding acquisition, Resources, Visualization, Writing – original draft. SG: Data curation, Investigation, Methodology, Writing – original draft. WL: Data curation, Methodology, Resources, Writing – original draft. YZ: Investigation, Project administration, Validation, Writing – original draft. HX: Conceptualization, Funding acquisition, Methodology, Project administration, Resources, Supervision, Validation, Writing – review & editing. JY: Conceptualization, Investigation, Methodology, Project administration, Resources, Supervision, Visualization, Writing – review & editing.
